# Analysis of the causes of failure after radical surgery in patients with _P_T_3_N_0_M_0_ thoracic esophageal squamous cell carcinoma and consideration of postoperative radiotherapy

**DOI:** 10.1186/s12957-017-1259-4

**Published:** 2017-10-25

**Authors:** Wen-Bin Shen, Hong-Mei Gao, Shu-Chai Zhu, You-Mei Li, Shu-Guang Li, Jin-Rui Xu

**Affiliations:** 1 Department of Radiation Oncology, The Fourth Hospital of Hebei Medical University, No. 12 Jiankan Road, Chang’an District, Shijiazhaung, 050011 China; 2 Department of Radiation, The First Hospital of Shijiazhaung, Shijiazhaung, 050011 China

**Keywords:** Esophageal cancer, Surgical treatment, Recurrence, Prognosis, Postoperative radiotherapy

## Abstract

**Background:**

Five-year overall survival rate of TESCC after surgery is low (approximately 30% to 60%), so it is meaningful to discuss the significance of PORT.

**Methods:**

We retrospectively collected the data of 227 patients with _P_T_3_N_0_M_0_ esophageal cancer (EC). The failure pattern after surgery was analyzed. Difference of adjuvant PORT in patients with _P_T_3_N_0_M_0_ TESCC and the appropriate population were explored based on the relevant studies.

**Results:**

There were 58 cases with intrathoracic locoregional recurrence (LRR) after radical surgery and 27 cases with distant metastasis, including 10 cases of recurrence. The recurrence rate of mediastinal lymph nodes in the thoracic cavity was 50.0%. Univariate analysis revealed that compared with patients with middle and lower thoracic EC, the 3/5-year survival rate of patients with upper thoracic EC was significantly lower, accompanied with remarkably higher thoracic LRR. Compared with those with moderately- and well-differentiated TESCC, the 3/5-year survival rate of patients with poorly differentiated TESCC was significantly lower, whereas the distant metastasis rate was notably higher. Multivariate analysis revealed that different lesion locations and different pathologic differentiation were the independent prognostic factors. The lesion location and degree of differentiation were the independent influencing factors for thoracic LRR and distant metastasis, respectively.

**Conclusion:**

The intrathoracic LRR is the major failure pattern for patients with _P_T_3_N_0_M_0_ TESCC after conventional two-field lymphadenectomy. In addition, recurrence rate of _P_T_3_N_0_M_0_ TESCC was significantly higher in upper thoracic EC than in middle and lower thoracic EC. PORT is recommended to patients with _P_T_3_N_0_M_0_ upper TESCC.

## Background

Esophageal squamous cell carcinoma (ESCC) is a common type of esophageal carcinoma (EC), which is characterized by rapid development and fatal prognosis in most patients. Radical esophagectomy (RO) is the mainstay of treatment for _P_T_3_N_0_M_0_ thoracic esophageal squamous cell carcinoma (TESCC) [1, 2]. Five-year overall survival (OS) rate after surgery is approximately 30 to 60%, whereas the recurrence rate is as high as 43% after RO [1–5]. Notably, the recurrence is the major cause of treatment failure in patients with _P_T_3_N_0_M_0_ TESCC.

In China, it has been reported that adjuvant postoperative radiotherapy (PORT) or PORT combined with postoperative chemotherapy (POCT) contributed to the reduction of postoperative recurrence in clinical practice [1, 6]. Currently, few literatures reported whether adjuvant PORT or combined RCT can reduce recurrence rate of _P_T_3_N_0_M_0_ TESCC. Yang et al. [7] reported that compared with surgery alone, postoperative assisted three-dimensional conformal radiotherapy (3D-CRT) decreased postoperative recurrence rate of _P_T_3_N_0_M_0_ TESCC and increased the 5-year disease-free survival (DFS) and OS. Thus, adjuvant PORT was recommended for patients with _P_T_3_N_0_M_0_ TESCC.

It is of great significance to understand the causes and patterns of treatment failure in postoperative patients with EC to further clarify the recurrent sites of patients and guide their follow-up treatment. To pinpoint the influencing factors of postoperative recurrence of _P_T_3_N_0_M_0_ TESCC, we investigated the failure pattern of _P_T_3_N_0_M_0_ TESCC and explored the extent and feasibility of irradiation target after surgery.

## Methods

### Patients

From January 2007 to December 2010, we collected the data of 227 patients (160 males and 67 females) aged from 43 to 79 with a median age of 62 who received esophagectomy in the Department of Thoracic Surgery, the Fourth Hospital of Hebei Medical University. The number of patients with upper, middle, and lower thoracic EC was 33, 153, and 41, respectively. Surgical procedure consisted of left (*n* = 212) and right (*n* = 15) thoracic esophagectomy. The median length of esophageal lesions was 5.0 cm (range 0.8–10.0 cm). The dissected lymph node numbers ranged from 5 to 28, with a median number of 10. Contrast-enhanced chest CT scan showed mediastinal lymph nodes with short axis diameter < 10 mm in 44 patients preoperatively. Intraoperative study found the presence of moderate to severe inflammatory adhesions between the esophagus and peripheral tissues and organs due to esophageal lesions in 97 cases, while mild adhesions or without adhesions in 130 cases. The procedures were approved by the Ethics Committee of the Fourth Hospital of Hebei Medical University. All patients signed informed consent. Needle aspiration biopsy was used for the enlarged lymph nodes in the superficial areas, such as supraclavicular area and neck. The diagnosis of mediastinum intrathoracic and celiac lymph nodes is based on the criteria in previous reports. The diagnosis of anastomotic recurrence is based on electronic gastroscopy and pathological biopsy.

### Inclusion and exclusion criteria

Inclusion criteria are as follows: (1) patients who received the total esophagectomy + two-field (complete mediastinal + abdominal) lymph node dissection by means of thoracotomy on both left and right sides in the Department of Thoracic Surgery, the Fourth Hospital of Hebei Medical University; (2) patients undergoing RO; (3) patients with postoperative pathological diagnosis of TESCC; and (4) patients with pathological stage _P_T_3_N_0_M_0_. Exclusion criteria are as follows: (1) patients with non-squamous cell carcinoma (SCC); (2) patients with no prior adjuvant PORT and/or postoperative chemotherapy (POCT); (3) patients with positive esophageal stump; and (4) patients with perioperative death and lack of follow-up data.

### Diagnostic criteria of treatment failure

Location of treatment failure and time were confirmed by reviewing data of patients with regular visits in our hospital, including surgical records and imaging reports (CT, MRI, and ECT), as well as pathological and cytological reports.

### Recurrence type

*Intrathoracic locoregional failure (LRF)* refers to mediastinal lymphatic metastasis, recurrence of the original esophageal neoplasms, and anastomotic recurrence. According to the seventh edition (2009) of the Union for International Cancer Control (UICC) on Cancer TNM staging system, LRF included the supraclavicular lymph node metastasis (SLNM) and abdominal lymph node metastasis (including left gastric and hepatic and splenic hilar lymph nodes as well as those around the celiac artery). *Distant metastasis* refers to hepatic, pulmonary, osseous, subcutaneous, and pleural metastases as well as metastasis to other parts of the body (such as armpit and inguina).

### Diagnostic criteria of postoperative lymphatic metastasis/recurrence

Enlarged superficial lymph nodes were mainly confirmed by cytology examination or postoperative pathology. CT diagnostic criteria of the lymph node in other areas are as follows: (1) short axial diameter of lymph node ≥ 10 mm, as compared with standard axial diameter ≥ 3 mm and ≥ 5 mm in special parts of the body, such as the paraesophageal area and tracheoesophageal groove; (2) small lymph nodes with irregular shape and low density necrosis at the center; (3) clustering of lymph nodes (*n* ≥ 3); and (4) lack of boundaries between lymph node and peripheral fat due to extracapsular invasion.

### Follow-up

The followed-up period of all patients were ended until December 31, 2015. Patients were instructed to return regularly for follow-up every 3–6 months for the first year and 6 to 12 months in the following years. Review items involved neck and abdomen ultrasound and contrast-enhanced chest CT/MR as well as ECT and PET CT examination when necessary. In terms of suspected cervical lymph node enlargement by ultrasound, needle aspiration biopsy or contrast-enhanced CT would be recommended, whereas suspected lymphadenopathy by abdominal ultrasound required contrast-enhanced abdominal CT/MR examination. The number of patients that followed up for 1, 3, and 5 years was 193, 136, and 109, respectively, and a total of 131 patients died at the last follow-up.

### Statistical analysis

We used SPSS 19.0 for the data analysis. The comparison of counting data was performed using chi-square test. Kaplan-Meier method was utilized to calculate OS rate, recurrence rate, and distant metastasis rate. Log-rank method and Cox regression model were applied to univariate analysis and multivariate analysis of the prognostic factors, respectively. Test level was α = 0.05.

## Results

### Analysis of failure pattern in the whole group

In the last follow-up, there were a recurrence of intrathoracic locoregional in 58 patients (LRR; recurrence rate of 25.6% and median recurrence time of 16.4 months, ranging from 2.2 to 81.9 months), distant metastasis in 27 patients (metastasis rate of 11.9% and median time of 24.5 months, ranging from 2.0 to 66.0 months), and recurrence with distant metastases in 10 patients. In 58 patients with recurrence, the number of supraclavicular, intrathoracic, abdominal, and anastomotic lymph node recurrence was 12, 39, 10, and 6, respectively, as well as the recurrence in tumor site in five patients (Table 1). The number of intrathoracic LRR in patients with upper, middle, and lower thoracic EC was 12, 38, and 8, respectively. The recurrence rate of abdominal lymph nodes was 9.8% in the patients with lower thoracic EC, which was higher than upper thoracic EC (0%) and middle thoracic EC (3.9%). There was no significant difference among upper, middle, and lower thoracic portions (χ^2^ = 4.393, *P* = 0.111). The SLNM in patients with upper thoracic EC was 12.1%, which was higher than middle thoracic EC (4.6%) and lower thoracic EC (2.4%); there was no significant difference among upper, middle, and lower thoracic portion (χ^2^ = 4.532, *P* = 0.104, Table 2).

**Table 1:** Recurrent sites of 58 cases with intrathoracic LRR

Recurrent site	No.	Rate (%)	Recurrent site	No.	Rate (%)
Sup.LN	6	10.3	Sup. LN+Med.LN+Abd. LN	1	1.7
Med.LN	29	50	Ana.	2	3.4
Abd. LN	4	6.9	Ana.+Med. LN	3	5.2
Sup. LN+Med.LN	3	5.2	Ana. +Abd. LN	1	1.7
Sup..LN+Abd. LN	2	3.4	Tumor site	4	6.9
Med.LN+Abd. LN	2	3.4	Tumor site +Med. LN	1	1.7

Note: Sup: supraclavicular; Med.: mediastinal; Abd.: abdominal; Ana.: anastomotic; LN: lumph node

**Table 2:** Analysis of recurrence pattern of different lesion locations N (%)

	Total No.	Upper thoracic portion(33)	Middle thoracic portion (153)	Lower thoracic portion(41)	Χ^2^	*P*
Sup.LN	12	4(12.1%)	7(4.6%)	1(2.4%)	4.532	0.104
Med.LN	39	9(27.3%)	26(17.0%)	4(9.8%)	3.954	0.138
Abd. LN	10	0(0%)	6(3.9%)	4(9.8%)	4.393	0.111
Ana.	6	1(3.0%)	3(2.0%)	2(4.9%)	1.092	0.579
Tumor site	5	0(0%)	4(2.6%)	1(2.4%)	0.874	0.646

Note: Sup: supraclavicular; Med.: mediastinal; Abd.: abdominal; Ana.: anastomotic; LN: lumph node. Overlapped recurrence pattern led to double counting of the number of patients with different recurrent sites (that is, the cumulative cases)

Among 27 patients with distant metastases, there were 16 patients with lung metastases (12 patients with simple lung metastasis, and 4 patients recurred accompanied with lung metastasis), 4 patients with liver metastasis (3 patients with simple liver metastasis, and 1 patient recurred accompanied with liver metastasis), 4 patients with bone metastasis (2 patients with simple liver metastasis, and 2 patients recurred accompanied with bone metastasis), 2 patients with brain metastases, and 5 patients with multiple metastases.

### Univariate analysis of the prognosis

**Fig. 1:**
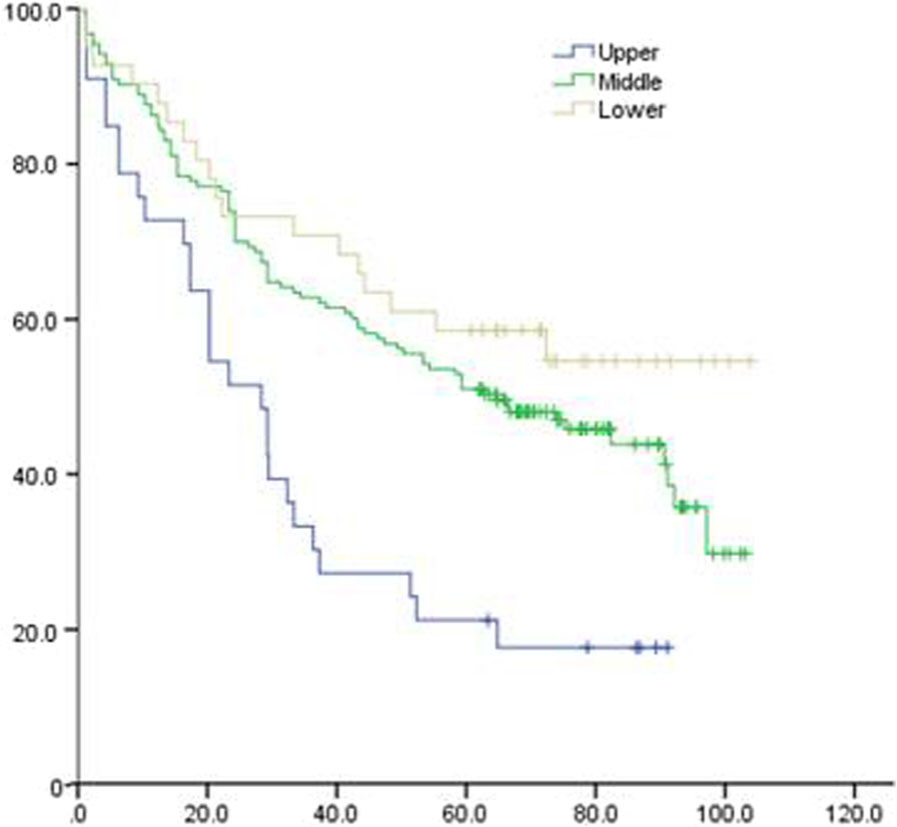
OS of different lesion locations

**Fig. 2:**
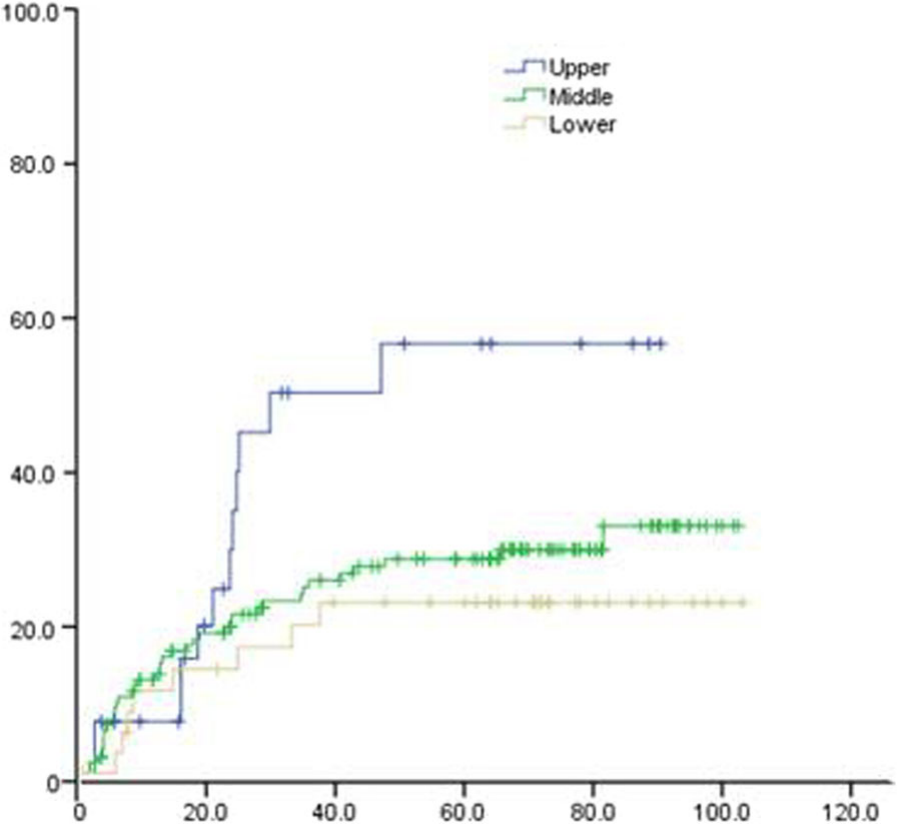
Thoracic LRR of different lesion locations

**Fig. 3:**
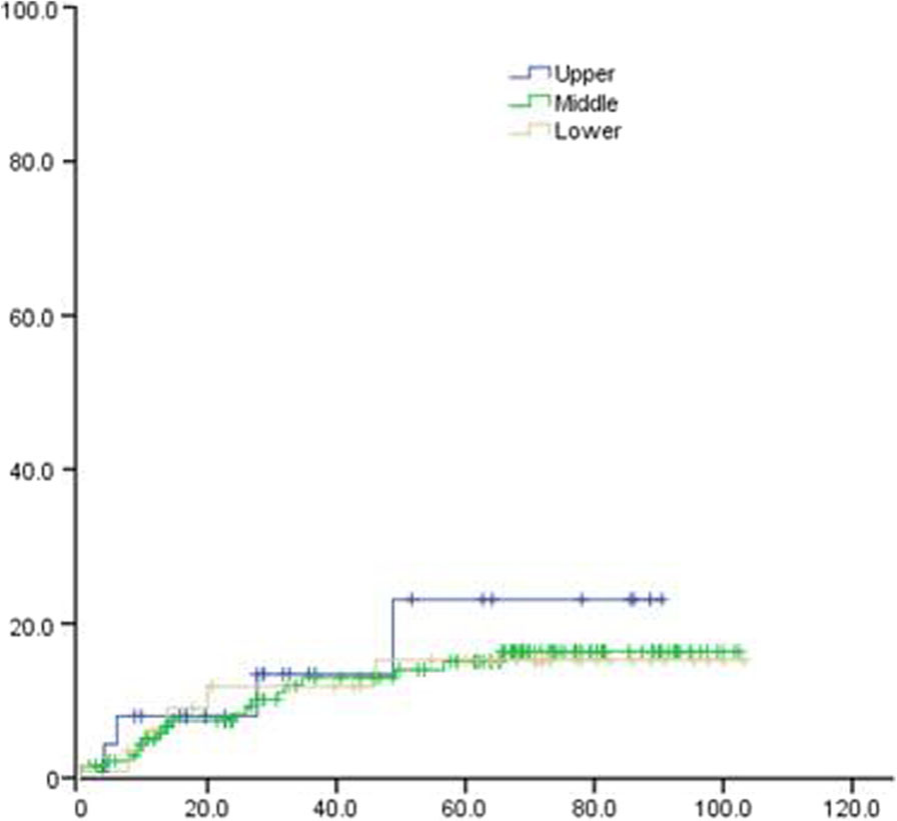
Distant metastasis rate of different lesion locations

**Fig. 4:**
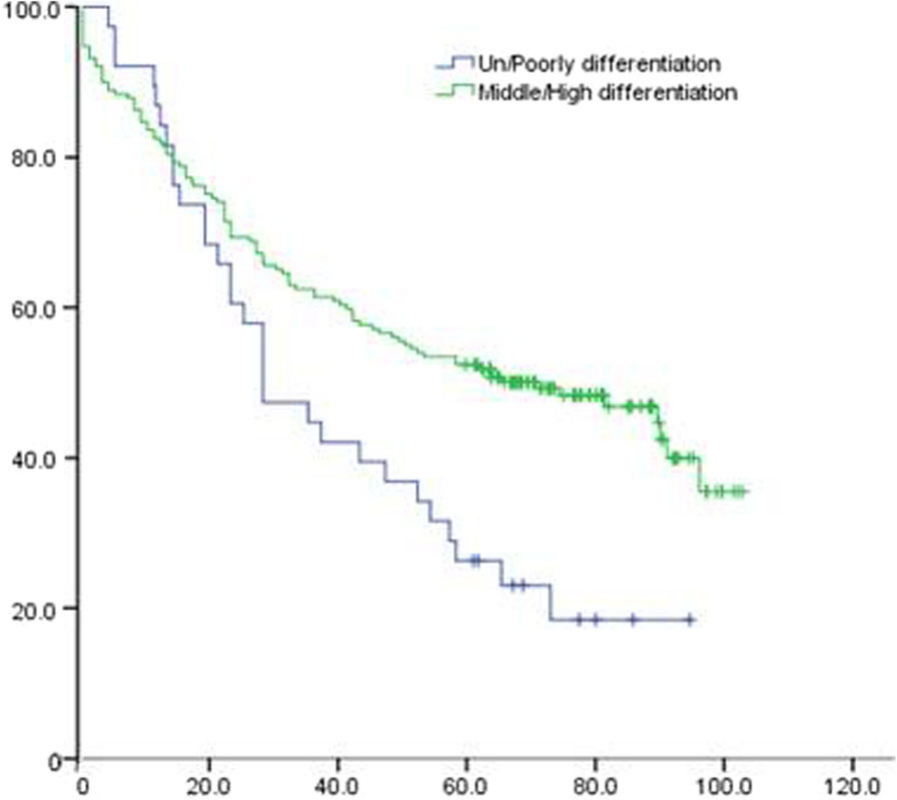
Survival rate of different degrees of pathological differentiation

**Fig. 5:**
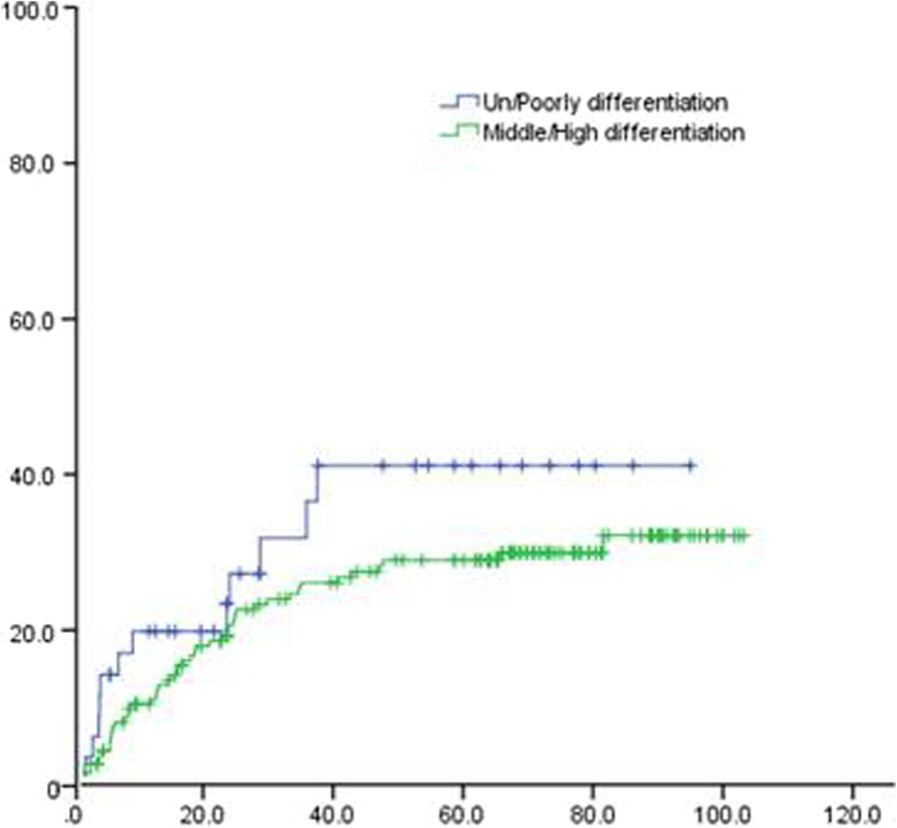
Thoracic LRR of different degrees of differentiation

**Fig. 6:**
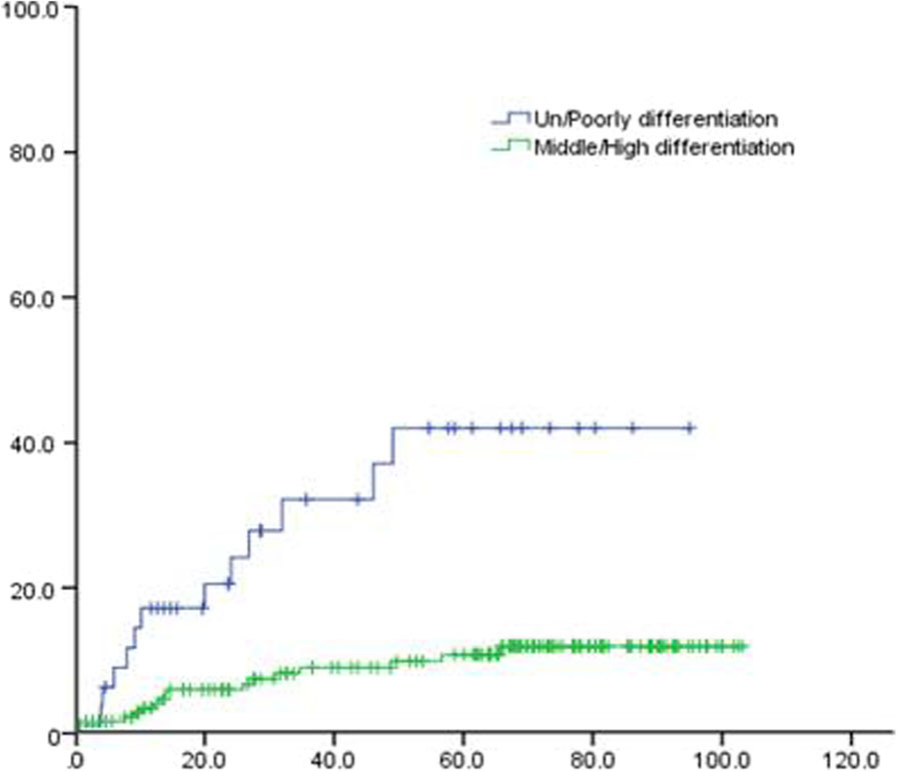
Distant metastases of different differentiations

Univariate analysis of the clinical and pathological data of patients showed that 3- and 5-year OS of patients with upper thoracic EC was significantly lower than that in patients with middle and lower thoracic EC (χ^2^ = 15.747, *P* = 0.000). The 3- and 5-year OS of poorly differentiated SCC patients was remarkably lower than that of moderate- and well-differentiated SCC (χ^2^ = 7.798, *P* = 0.005). The 3- and 5-year distal metastatic rate of poorly differentiated SCC was notably higher, compared with moderate- and well-differentiated SCC (Χ2 = 19.243, *P* = 0.000). The 3- and 5-year thoracic LRR rate of patients with upper thoracic EC was significantly higher than that in middle and lower EC patients (χ^2^ = 5.923, *P* = 0.047) (Figs. 1, 2, 3, 4, 5, and 6 and Table 3).

**Table 3:** Univariate analysis of prognosis of 227 patients with _p_T_3_N_0_ M_0_ TESCC

Variable	N	OS (%)		Χ^2^	*P*	LRR (%)		Χ^2^	*P*	DM (%)		Χ^2^	*P*
		3yr	5yr			3yr	5yr			3yr	5yr		
Sex				1.819	0.177			0.985	0.321			2.301	0.129
Male	160	58.1	45.6			22.4	27.1			24.1	18.2		
Female	67	62.7	53.7			32.8	34.7			7.4	7.4		
Age				0.513	0.474			0.788	0.375			0.172	0.678
≤62	120	59.2	48.3			27.6	32.5			14.7	16.0		
>62	107	59.8	47.7			24.1	26.7			9.0	13.7		
Lymph node by preoperative CT				1.326	0.250			0.395	0.530			0.013	0.908
No	153	58.2	46.4			26.1	29.8			10.2	14.7		
Yes	44	50.0	40.9			31.9	31.9			16.2	16.2		
Lesion location				15.747	0.000			5.923	0.047			0.378	0.828
Upper	33	30.3	21.2			49.2	55.6			12.6	22.3		
Middle	153	62.7	51.0			24.0	27.7			12.1	14.2		
Lowe	41	70.7	58.5			19.2	22.0			11.0	14.4		
Lesion length(cm)				0.413	0.520			0.098	0.754			0.215	0.643
≤5	125	55.2	47.2			27.0	30.5			13.4	14.8		
>5	102	64.7	49.0			24.9	29.0			10.0	14.8		
Intraoperative adhesion				0.487	0.485			0.003	0,957			0.237	0.627
No/mild	130	60.0	49.2			25.6	29.8			12.0	15.8		
Moderate/severe	97	58.8	46.4			26.6	29.7			12.0	13.8		
Differentiation				7.798	0.005			1.563	0.211			19.243	0.000
poorly	38	44.7	26.3			30.8	40.0			31.1	40.9		
Moderately to well	189	62.4	52.4			25.0	27.9			8.0	9.7		
No. of lymph nodes removed(No.)				2.422	0.120			0.001	0.978			0.007	0.935
≤10	133	57.1	43.6			26.9	30.3			12.1	14.9		
>10	94	62.8	54.3			24.9	29.1			11.7	14.9		

### Multivariate analysis of the prognosis of patients

The influencing factors for prognosis of patients were put into the Cox model for multivariate analysis. The results revealed that the degree of differentiation and lesion location were independent prognostic factors for OS of patients (*P* = 0.014, 0.010). Lesion location was the independent influencing factor for thoracic LRR (*P* = 0.46). The degree of differentiation was the independent influencing factor for distant metastasis (*P* = 0.000) (Table 4).

**Table 4:** Multivariate analysis of prognosis in 227 patients with _p_T_3_N_0_M_0_ TESCC

Variable	B	SE	Wald	*P*	OB	95%CI	
						Lower	Upper
**OS**							
Differentiation	0.429	0.174	6.082	0.014	0.651	0.463	0.916
Lesion location	0.567	0.221	6.594	0.010	0.567	0.368	0.874
**LRR**							
Lesion location	0.498	0.247	3.997	0.046	0.598	0.362	0.990
**DM**							
Differentiation	1.727	0.421	16.825	0.000	0.178	0.078	0.406

## Discussion

In the present study, patients with _p_T_3_N_0_M_0_ TESCC undergoing surgery alone were followed up for 5 years. Among them, 58 patients experienced intrathoracic LRR, with a recurrence rate of 25.6%. Most patients recurred in 1 to 2 years after surgery, with a median recurrence time of 16.4 months. The median survival time of patients after recurrence was 5.6 months, ranging from 0.6 to 46.7 months. The most common area for recurrence is the thorax, specifically the mediastinal lymph nodes. The results showed that simple mediastinal lymphatic recurrence rate was as high as 50.0% (29/58), and the recurrence rate of other areas was less than 10%. These findings suggested that major postoperative failure for pT_3_N_0_M_0_ TESCC patients was attributed to mediastinal lymphatic recurrence. This was consistent with the results of previous studies. Yang et al. [7] compared surgery alone and PORT in 916 patients with pT_2-3_N_0_M_0_ TESCC. Wang et al. [8] analyzed failure pattern of 208 patients with _p_T_3_N_0_M_0_ TESCC, and the results indicated a LRR rate of 25.0% and the mediastinal lymph nodes being the most common recurrent site, with a median recurrence time of 15.5 months. Zhang et al. [9] reported that a total of 467 patients met the inclusion criteria (including 228 _p_T_3_N_0_M_0_ patients) according to the analysis of survival factors affecting stage II EC patients. Therefore, we propose that in terms of postoperative adjuvant therapy for patients with _p_T_3_N_0_M_0_ TESCC, the mediastinal lymph node is the primary focus.

Appropriate prophylactic PORT in theory can reduce the recurrence rate of mediastinal lymph nodes in the thoracic cavity, thereby improving OS of patients. However, in the era of two-dimensional radiotherapy (2D-RT), PORT for the lymph node-negative patients with EC mainly led to unsatisfactory improvement of their survival. For example, Xiao et al. [4] indicated that compared with those undergoing surgery alone, 3-year OS of patients with _p_T_3_N_0_M_0_ EC receiving PORT was increased by 8%; there was no difference in 5-year OS. Chen et al. [1] found patients with negative lymph nodes confirmed by postoperative pathology did not benefit from PORT, and advances in 3D-CRT and intensity-modulated RT contributed to the rapid development of adjuvant PORT for EC.

In recent years, the application of 3D-CRT to adjuvant therapy for _p_N_0_ EC has been reported, which was of great significance to clinicians. Yang et al. [10] demonstrated that the 5-year OS and progression-free survival rates were 74 and 71%, assuming that 3D-CRT was safe and effective for postoperative patients with pT2-3N0M0 EC, and the recurrence rate and survival rates were higher than previously reported in the literature. We deem that 3D-CRT is advantageous over 2D-RT in the target dose distribution and normal tissue volume. However, the actual survival benefit should be further validated with more cases accumulated.

There is no international consensus as to the role of adjuvant PORT for EC, which may be related to inclusion criteria, surgical methods, irradiation mode, extent of exposure, and radiotherapy technologies used in different research centers. Currently, stratification analysis is recommended in most clinical studies. Therefore, postoperative adjuvant therapy may produce different results in patients with _p_T_3_N_0_M_0_ TESCC, thus stratification analysis was performed on all patients enrolled. We found that the lesion location was the independent influencing factor for OS and recurrence of _p_T_3_N_0_M_0_ TESCC patients. OS was significantly lower in patients with upper thoracic EC than those with middle and lower thoracic EC, accompanied with notably higher recurrence, which is in accordance with most of the previous reports [11]. This may be related to the different anatomical structures of different lesions. The higher location of the upper thoracic lesions leads to incomplete removal of lymph nodes in the corresponding areas [12]. This may also be associated with left thoracic RO and two-field (thorax+abdomen) lymphadenectomy. Limited surgical field of vision led to insufficient removal of mediastinal lymph nodes in drainage area, giving rise to high recurrence rate in this area. However, other studies reported lesions were not related to postoperative recurrence of EC. For example, Liu et al. [13] reported different lesions were not significantly associated with lymph node recurrence. Based on our findings, we recommend PORT to patients with _p_T_3_N_0_M_0_ thoracic EC after thoracic and abdominal lymph node dissection.

In this study, the removed lymph node median number in EC patients was 10, which was less than Western countries [14–16]. Chen et al. [17] suggested that the removed lymph node number < 6 might lead metastatic lymph node omission, thus affecting the accuracy of the *N* grade. According to UICC, the number of surgically removed lymph nodes is reasonably suggested to be no less than six. We believe that the more thorough lymph nodes dissected intraoperatively, the less possibility of lymph node metastasis, thereby reducing the LRR rate of patients and improving the survival rate of patients. Nonetheless, further study is needed to explore whether the benefit brought by lymph node dissection is offset by its negative effect on the patient’s immunity. The prognostic factors that affect the distant metastasis in this group were shown to be closely associated with the degree of disease differentiation. Additionally, consistent with the conclusions of most studies [5, 13, 18], the distant metastasis rate of poorly differentiated EC was significantly higher than that of moderately and well-differentiated EC. Advances in comprehensive treatments and their increased application in clinical treatment of EC have gradually established the standard treatment of postoperative EC using PORT combined with POCT. Postoperative radiochemotherapy (PORCT) can improve the long-term survival of EC patients [19], but further studies are required to confirm whether PORCT can effectively reduce distant metastasis rate of the _p_T_3_N_0_M_0_ TESCC, thereby improving the survival rate.

## Conclusion

In summary, the results of the present study reveal that intrathoracic LRR of _p_T_3_N_0_M_0_ TESCC after conventional two-field (thoracic and abdominal) lymphadenectomy contributes to treatment failure, and most of the recurrence occurs within 2 years after surgery. The lesion is the main influencing factor for prognosis and recurrence, and the recurrence rate of patients with upper thoracic EC was significantly higher than that in the middle and lower thoracic EC. Therefore, PORT is strongly recommended for _p_T_3_N_0_M_0_ TESCC patients to reduce postoperative recurrence rate of mediastinal lymph nodes in thoracic cavity. For patients with poorly differentiated EC, selection of CT needs further confirmation.
